# Mechanical and aging performance of natural fiber-reinforced mycelium composites for sustainable packaging

**DOI:** 10.1038/s41598-026-49585-x

**Published:** 2026-04-18

**Authors:** Prasanna C. Kattimani, Akash Biradar, Yashwant Munde, Rayappa Shrinivas Mahale, Ravi Shankar Rai, Ananda Hegde

**Affiliations:** 1 Department of Automation & Robotics Engineering, Army Institute of Technology, Pune, Maharashtra India; 2 Department of Mechanical Engineering, Dr. D. Y. Patil Institute of Technology, Pune, Maharashtra India; 3 Department of Mechanical Engineering, MKSSS’s Cummins College of Engineering for Women, Pune, Maharashtra India; 4 Department of Automation and Robotics, JSPM’s Rajarshi Shahu College of Engineering, Pune, Maharashtra India; 5 Manipal Institute of Technology, Manipal Academy of Higher Education, Manipal, Karnataka India

**Keywords:** Mycelium-based composites, Sustainable packaging, Biodegradable materials, Mechanical properties, Natural fibres, Biotechnology, Engineering, Materials science

## Abstract

The increasing demand for sustainable and biodegradable alternatives to petroleum-based packaging materials has stimulated growing interest in mycelium-based bio-composites. In the present work, natural fiber-reinforced mycelium composites were developed and evaluated with emphasis on their mechanical performance and aging behavior for packaging applications. Two composite systems Jute/Rice Straw/Mycelium (JF/RS/M) and Jute/Cocopeat/Mycelium (JF/CP/M) were fabricated under controlled growth conditions to investigate the influence of reinforcement type and growth duration on mechanical properties. Compressive and flexural tests were conducted after 15, 25, 35, and 60 days of growth to characterize strength evolution and structural stability. The results indicate a pronounced increase in mechanical performance with increasing mycelial colonization, with peak properties observed at 25 days. Significant enhancements in mechanical performance were observed relative to the initial testing stage, with compressive strength increasing by 50.3% for JF/RS/M and 69.9% for JF/CP/M composites, and flexural strength improving by 68% and 106%, respectively. To assess aging behavior, mycelial growth was terminated after 35 days, and mechanical testing at 60 days confirmed the retention of structural integrity with no significant degradation in strength. The novelty of this study lies in systematically evaluating the influence of mycelial growth duration on the mechanical evolution and aging stability of natural fiber–reinforced mycelium composites, providing insight into their suitability for sustainable packaging applications.

## Introduction

 Plastic has transformed the manufacturing industry; however, the effect caused by this progress affects industries in contemporary society. To date, 91% of plastics obtained in the world remains non-recycled, indicating that people ingest 5 g of micro plastics in their food on a weekly basis^1^. Mycelium technology may represent one of the viable solutions, such as this mycelium brick, which functions as a plastic-like substitute offering various uses and fresh opportunities for products, perhaps even in the realm of wearable technology^2^. It is very much possible that the mycelium technology can help achieve a more renewable and cleaner future. Plastics exist in a variety of types, shapes, colours, and sizes, with soda bottles being a notable example. Nevertheless, from the perspective of a chemist, they are all classified under the same group of materials: polymers. While Bakelite, recognized as the first synthetic plastic made from organic compounds, was invented in 1907, the identification of polymers took place in 1920 by Hermann Staudinger. The term polymer is a comprehensive designation for substances with large molecules formed from repeating subunits that are chemically bonded together. World War II acted as a major driving force behind the evolution of plastic and chemical innovations, including Polyethylene, Polystyrene, and Nylon. This reflects a rather grim facet of innovation, where warfare can hasten development. In the following decade of the 1950s, plastic manufacturers began mass-producing consumer goods to capitalize on the materials they had created during war times^3^. The organism has a root-like part called the mycelium, which is the main part of fungi responsible for the production of mushrooms. The mycelium acts like roots in the organism because it spreads in the soil in search of nutrients, while mushrooms are responsible for reproduction just like flowers^4^. The mushrooms we eat are just a small, visible part of a much larger organism. Fungi are incredibly important in ecosystems because they are able to recycle nutrients, making nutrients that might otherwise be unavailable to other organisms, such as plants, become available. Under the right conditions, they can be amazingly resilient and spread with ease. Just a few spores is all it takes to begin germination. As the fungi grow, they excrete enzymes, breaking down their surroundings to uptake nutrients. As the cells begin to branch out and continue to grow, a large network of mycelium develops, and it isn’t until the mycelial network has fully developed that mushrooms begin to appear.

Rather than allowing the mushrooms to grow naturally, it is possible to grow the structure around the mycelium, which would enable the production of predictable shapes. This production process is extremely simple^5^. It employs a number of agricultural waste materials that could be hemp to wood chips. It is combined with mycelium matrices. Mycelium foam is essentially the basic ingredient for most mycelium products. It is poured into a cast and set in a rigorous environment with a regulated level of CO2, moisture content, air flow, and temperature. It is a very fast procedure where fibers show appearance in a few hours with a layer evident in a day or two at best. Typically, a week is enough for the mycelium foam completely filling the cast. Typically, the design takes about a week to complete. Mycelium foam is a great insulator, but it is strong, non-toxic, robust, and biodegradable thus opening a wide range of applications for its use in packaging materials, clothing, construction materials, or even food items. While plastics or other synthetic materials take centuries to biodegrade, mycelium-based materials biodegrade once their shelf life is over^6^. One of the key advantages of mycelium foam is its cost-effectiveness, as it is economically competitive with conventional materials such as polystyrene foam.

Mycelium technology has given rise to the existence of many startups and organizations across various industries globally^7^. With over 40 patents across 31 different countries, it is safe to say that the majority of mycelium-based materials and composites are produced through the licenses offered by them. Ecovative has created various lines of products, and the application of the ‘MycoFlex’ technology is used to develop everything from insulating lofts for gloves to the use of foams in footwear^8^. This is heat-resistant, insulates well, is breathable, and resilient^9^. The needs of the packaging industry include the need for high-performance, cost-competitive packaging technology which meets the requirements of thermal insulation, is waterproof, and biodegradable in soil in 45 days^9^. Within the packaging sector, there is a growing demand for high-performance and cost-effective materials that provide thermal insulation and water resistance while also being capable of biodegrading in soil within approximately 45 days. This option is a superior alternative to polystyrene. Additionally, mushroom-based packaging consumes merely 12% of the energy used in plastic production and emits 90% less CO2 equivalents^10^. In the case of most commercial products, mycelium undergoes heating for an extended period prior to reaching the consumer. This process is essential to eliminate the mycelium, preserve the desired shape of the product, and inhibit the growth of mushrooms and the release of spores. Additionally, mycelium has found applications within the construction sector^11^. A notable example is the UK-based start-up Biohm. This company has been creating a mycelium insulation panel that is set to be the world’s first accredited mycelium insulation product. In addition to its health and safety benefits, mycelium excels beyond petrochemical and plastic-based construction materials regarding thermal and acoustic insulation^12^. Furthermore, in the event of a fire, mycelium does not produce harmful toxic smoke as it is not composed of synthetic, resin-based materials^13–15^. However, employing mushrooms as a load-bearing construction material necessitates significant research and development^16^. It is not as robust and lacks a long useful lifespan when compared to the majority of building materials. The global strategy for sustainable development seeks to reduce the reliance on non-renewable materials by replacing them with bio-based alternatives^17–23^. Recent studies have demonstrated the growing potential of natural fiber-reinforced composites for engineering applications. For instance, strategically interleaved continuous sisal fiber-reinforced 3D printed PLA composites The study developed 3D-printed PLA biocomposites reinforced with strategically interleaved continuous sisal fibers to improve mechanical and structural performance^24^. Similarly, bio-based materials have been explored in tribological applications such as Allium sativum-derived brake pads & the findings indicate that bio-derived additives can enhance tribological performance while supporting sustainable and environmentally friendly material development^25^. Hybrid natural fiber composites have also been explored to enhance the performance of sustainable materials. For example, pineapple and hemp fiber composites hybridized with glass fiber mats exhibited improved mechanical properties, achieving tensile strength of 58.24 MPa and flexural strength of 98.35 MPa, while tailored stacking sequences provided enhanced impact resistance for lightweight automotive panel applications^26^. Sustainable material development has also been demonstrated through green synthesis approaches, such as plant-mediated production of silver nanoparticles for environmental remediation. These studies reflect the growing emphasis on utilizing natural resources for eco-friendly material systems, supporting the development of bio-based composites such as mycelium-based materials^27^. Recent studies have demonstrated that agrowaste-based composites reinforced with nano-fillers such as nanoclay and SiO₂ can significantly improve mechanical performance and structural stability, highlighting the potential of agricultural residues as sustainable reinforcement materials. This supports the use of bio-based substrates such as rice straw and cocopeat in the development of mycelium-based composites^28^. Natural fiber-reinforced hybrid composites have shown significant improvements in mechanical and thermal performance when combined with suitable additives, as demonstrated in hemp/PP-based systems reinforced with silicon and zinc oxides. Such studies highlight the potential of bio-based reinforcements in developing sustainable composite materials with enhanced functionality^29,30^. Previous studies have shown that environmental factors such as moisture absorption can significantly influence the mechanical and viscoelastic properties of natural fiber composites, emphasizing the importance of durability assessment in sustainable material systems. This is consistent with the present study, which evaluates the aging behavior of mycelium-based composites^31^. Biomass-derived materials have gained significant attention for their potential in sustainable applications, including catalysis and waste management. Such developments highlight the versatility of natural resources and support the use of biomass-based composites, such as mycelium-derived materials, for environmentally friendly applications^32^. Biomass-derived materials have been shown to serve as sustainable and eco-friendly alternatives for advanced applications, highlighting the potential of bio-based resources in material development^33^.

In this aspect current research article investigates biodegradable alternatives to traditional packaging materials such as plastics and Styrofoam. Two types of biodegradable materials were developed: one composed of mycelium, rice straw, and jute, and the other made from mycelium, cocopeat, and jute. Jute fiber was selected as a reinforcement due to its high cellulose content and favorable mechanical properties, while rice straw and cocopeat were chosen as substrate materials owing to their lignocellulosic composition, which promotes effective mycelial growth and bonding. Furthermore, these materials are low-cost, abundantly available agricultural by-products, making them suitable for the development of sustainable and biodegradable composite systems. These materials were fabricated into samples by a mold with dimensions of 360 × 200 × 50 mm and then tested for their mechanical properties, focusing on flexural and compressive strengths-these are the major kinds of stresses that packaging materials usually face. The results were compared with the mechanical properties of traditional Styrofoam to determine the feasibility of mycelium-based materials as sustainable alternatives for packaging.

## Materials & methodology

### Materials

**Fig. 1:**
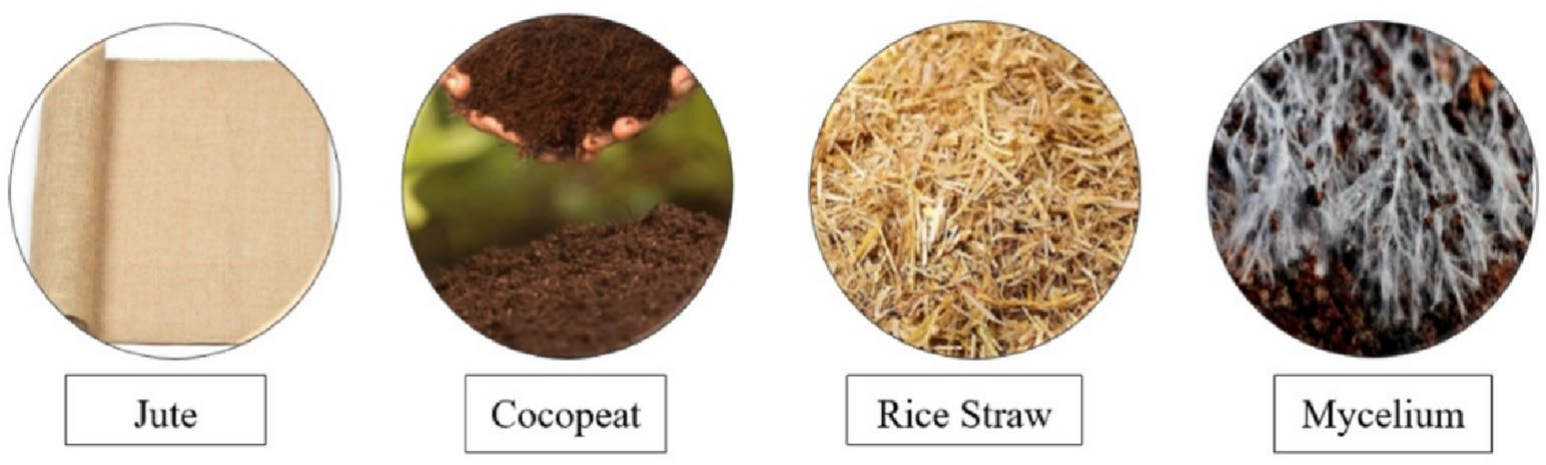
Materials used for the fabrication of mycelium-based bio-composite.

The materials employed in making mycelium-based bio composite materials are jute fibers, cocopeat, rice straw, and mycelium. The major components of jute fibers are cellulose and lignin, mainly obtained from the bast of plants such as kenaf, hemp, and flax. The high strength and biodegradability of this natural material make it quite popular in sustainable packaging materials. Cocopeat is a natural and biodegradable soil conditioner found to improve soil porosity and promote efficient root development of mycelium (Fig. 1).

Rice straw is a rice production waste and has excellent physical, thermal, and chemical properties. Mycelium biocomposites are constructed using agricultural waste and mycelium in molds, in which mycelium grows and binds the agricultural waste together. The result is then processed using high-temperature treatment after a predetermined period of growth, and this results in biodegradability and sustainability in place of Styrofoam packaging materials, which are biodegradable but not sustainable.

### Mycelium-based composite fabrication and growth on substrates

**Fig. 2:**
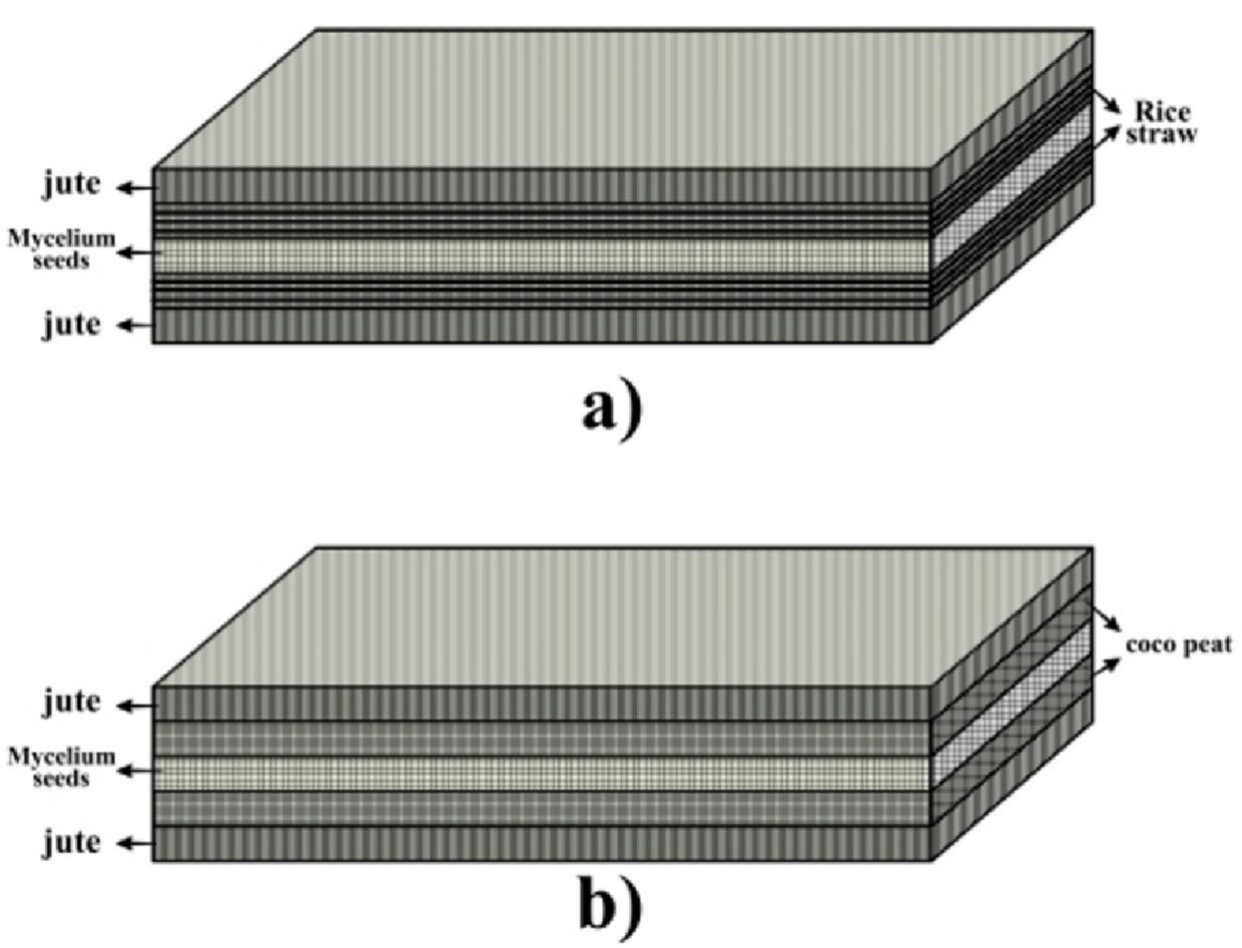
Schematic of the composite fabricated (**a**) jute + rice-straw + mycelium (JF/RS/M), and (**b**) jute + cocopeat + mycelium (JF/CP/M).

The materials used for preparing this study are rice straw, cocopeat, jute fibers, and mycelium seeds. Two different configurations of composites have been made. The first one includes rice straw, jute, mycelium, jute, and rice straw. This composite has been designed in a way that it looks like a sandwich, which can be observed from Fig. 2a. In the second one, the materials have been positioned in this manner: cocopeat, jute, mycelium, jute, and cocopeat, which can be viewed from Fig. 2b. The main purpose of processing these raw materials has been to test their effects regarding the strength of composites (Table 1).

**Table 1:** Composition of mycelium-based composites (mass fraction, wt%).

Component	JF/RS/M composite (wt%)	JF/CP/M composite (wt%)
Rice straw (RS)/cocopeat (CP)	49.5	49.5
Jute fiber (JF)	49.5	49.5
Mycelium spawn (M)	1.0	1.0
Total	100	100

The substrate materials were first arranged in the molds and sterilized at 80 °C for 60 min to eliminate contaminants. After cooling to room temperature for 24 h, mycelium spawn corresponding to 1 wt% of the total substrate weight was inoculated into the sterilized substrates before incubation. The seeds were cultivated in an environmental setup at 30 °C in the dark, aiming at facilitating development of mycelium. The incubation time for the seeds was varied to 15, 25, and 35 days in order to analyze/assess development of mycelium and bonding strength. To minimize microbial contamination, the substrates and molds were sterilized at 80 °C for 60 min prior to inoculation. The mycelium spawn was introduced under clean laboratory conditions, and the molds were sealed during incubation. The samples were periodically inspected for visible signs of contamination such as abnormal coloration, odor, or non-uniform fungal growth, and no contamination was observed during the incubation period. The tested mechanical properties were tended after making the composite materials. Compressive strength and flexural strength tests were performed to check the load-carrying capacity, bending strength, and interlayer bond strength of the biocomposites, respectively. Microstructural analysis was done to test the homogeneity of the mycelium growth. Standardized conditions were followed in the experiment, including the mycelium seed ratio of 1% (w/w), RH of 60%, and temperature of 30 °C. This research work is intended to compare the strength of the biocomposites made of rice straw and cocopeat in order to find the best combination to form high-quality biocomposites. After the completion of the growth period, the composites were removed from the incubation environment and oven-dried at elevated temperature to deactivate the mycelium, thereby preventing further biological activity and ensuring structural stability during testing.

### Three-point bend test

The flexural properties of the fabricated composites were studied with a 3-point bend test according to ASTM D790. It is one of the most common test methods for composite materials, and the ability to resist deformation under external loading conditions can be obtained from the result.

**Fig. 3:**
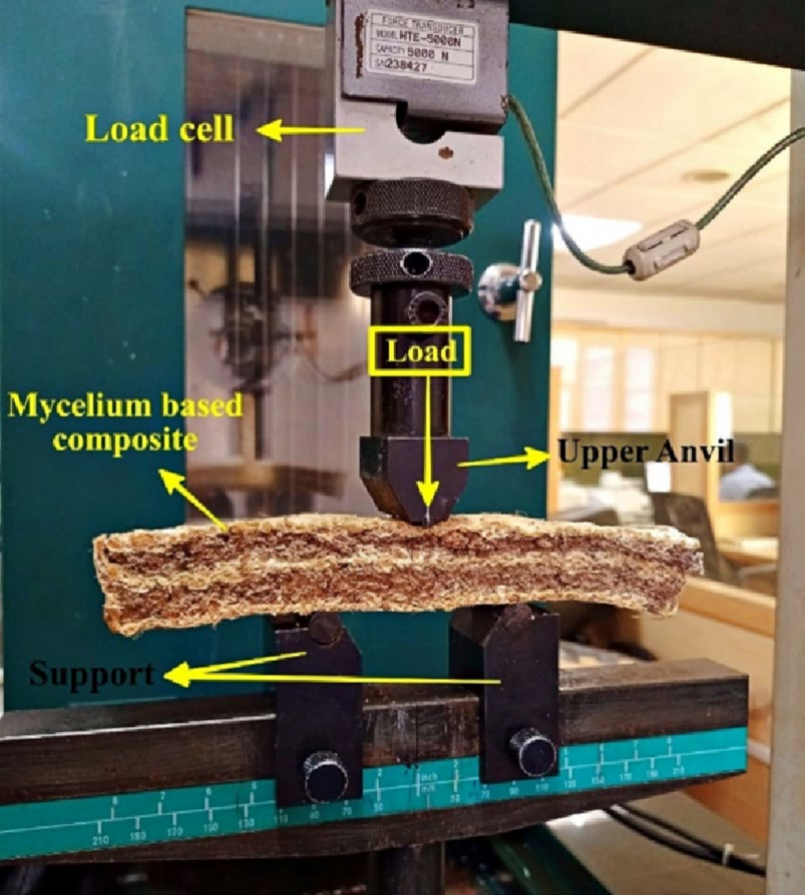
Three-point bend test on MBC sample.

The samples of specified dimensions (ref. Fig. 2) are placed horizontally and supported at two points. The bending load is applied at the centre from the upper cylinder as shown in Fig. 3. With increasing load, the composite sample experiences both compressive as well as tensile stresses. The force required to bend the sample until it fractures or till considerable deflection was determined.

### Compression test

**Fig. 4:**
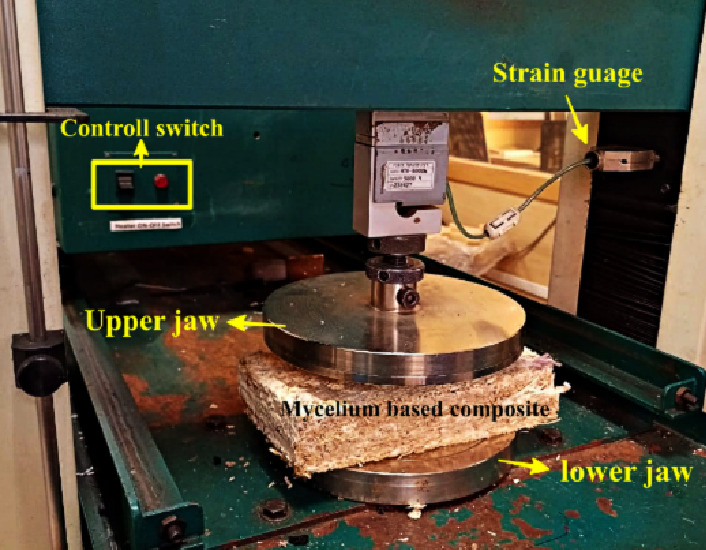
Compression test on MBC sample.

A compression test was conducted according to ASTM D6641 to determine the compressive properties of the fabricated MBC samples. The samples are prepared for the dimensions of 10 cm × 10 cm × 4 cm, and carefully placed on the lower jaw of the compression testing machine. During the compression test, the upper jaw moves to apply the compressive load gradually. The test is conducted at ambient atmosphere with a strain rate of 4 mm/min (Fig. 4).

Although ASTM D790 and ASTM D6641 standards were followed for testing methodology and loading conditions, the specimen dimensions were modified to accommodate the low-density and heterogeneous nature of mycelium-based composites. Larger specimens (10 cm × 10 cm × 4 cm) were used in compression testing to ensure structural stability and representative bulk behavior.

## Results and discussion

Recent advancements in composite materials research highlight the importance of integrating experimental and computational approaches to better understand material behavior^27^. Machine learning techniques have been increasingly employed for predictive modeling of thermoplastic nanocomposites, enabling accurate estimation of mechanical performance based on material parameters^34^. Similarly, multiscale modeling approaches have provided deeper insights into the structure–property relationships of natural fiber-reinforced composites^35^. In addition, studies on hybrid systems such as Innegra–hemp/epoxy composites have demonstrated the significant influence of environmental factors, including weathering, on long-term mechanical and viscoelastic performance^36^. These findings underline the need for further investigation into the durability and performance optimization of sustainable materials, such as mycelium-based composites, for practical applications. However, the influence of density and porosity on mechanical performance will be investigated in future studies.

### Morphological characterization

**Fig. 5:**
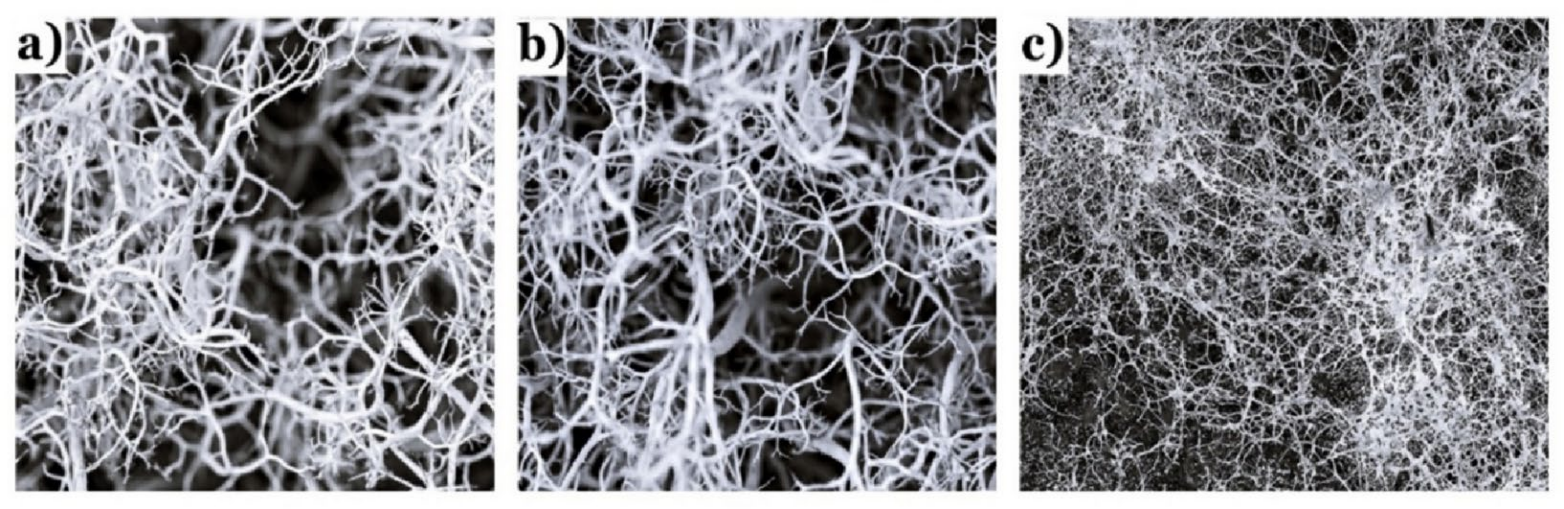
High-quality image of progression of mycelium growth after (**a**) 15 days, (**b**) 25 days, and (**c**) 60 days.

Figure 5 above showed the growth of the mycelium network for different periods, focusing on the impact that the mycelium network has on the composite strength. In Fig. 5a, the mycelium network has just started to grow, and root structures have started to develop in the substrate. At this point, the mycelium network has not grown to full maturity, and spaces between the fibres are evident. However, in Fig. 5b, after 25 days, the mycelium has reached full maturity, and the root structures have grown in size, occupying spaces in the composite, thus making the composite denser. At 60 days Fig. 5c, the mycelial network exhibits non-uniform characteristics, with certain regions showing reduced hyphal thickness and less dense connectivity. This indicates structural changes in the network after prolonged growth. Such variations may be associated with aging effects, including reduced moisture content and nutrient availability, leading to localized weakening of the bonding structure. These morphological changes correlate with the observed reduction in mechanical properties at extended durations^37^.

### Compressive strength

The values of the results for compression tests on RS/M/JF and CP/M/JF composites after 15 days of growth of mycelium were 145.3 kPa and 165.7 kPa, respectively, as taken from Table 2. However, on increasing the period of growth to 25 days, the value of the compressive strength of the composites remarkably enhanced to 230 kPa and 243 kPa, demonstrating an increment of 50.3% and 69.9% for RS/M/JF and CP/M/JF, respectively, from those values for which growth took 15 days. The considerable enhancement of the value of compressive strength for 25 days can certainly be attributed to the complete maturation of the mycelium network, which optimizes the interfacial bonding between the fibers and the mycelium matrix. The increased density of the mycelium and the root-like structure help to improve the densification of the composite to resist compressive loads. Additionally, the uniformity associated with the mycelial distribution provides a more even structure for the composite material, avoiding areas which might prove vulnerable to compressions.

**Table 2:** Results from compression testing of the fabricated composites.

Mycelium based composite	Growth duration	Compressive strength (kPa)				
		Sample 1	Sample 2	Sample 3	Average	Standard deviation
JF/RS/M	15 days	153.2	144.6	138.2	145.3	6.15
	25 days	227.8	232.4	230.6	230.3	1.89
	35 days	132.4	142.7	139.5	138.2	4.30
	60 days	125.6	128.4	132.5	128.8	2.83
JF/CP/M	15 days	178.6	167.2	151.3	165.7	4.53
	25 days	243.5	238.7	247.3	243.2	3.52
	35 days	204.3	210.5	198.4	204.4	4.94
	60 days	200.1	199.5	196.2	198.6	1.71

A slight reduction in the compressive strength was observed in both RS/M/JF and CP/M/JF composites after 35 days of growth. The reduction in the compressive strength beyond 35 days can be arrived at by a number of reasons:

1. Overgrowth & mycelium ageing: Overgrowth can also lead to a tangle of mycelium, hindering the structural integrity of the composite material as the mycelium deteriorates through ageing^38^.
2. Depletion of nutrients: When the mycelium starts increasing in size, it results in depletion of nutrients in the substrate, which makes it impossible for further structural development and reduces mechanical strength^39^.
3. Moisture-induced softening: Exposure of the composite to the growth environment may increase its moisture content, thereby diminishing the modulus of the composite^40^.
4. Degradation of fibres: The prolonged biological activities may weaken the reinforcing fibres to some extent, reducing their ability to resist the compression forces of the matrix^41^.

**Fig. 6:**
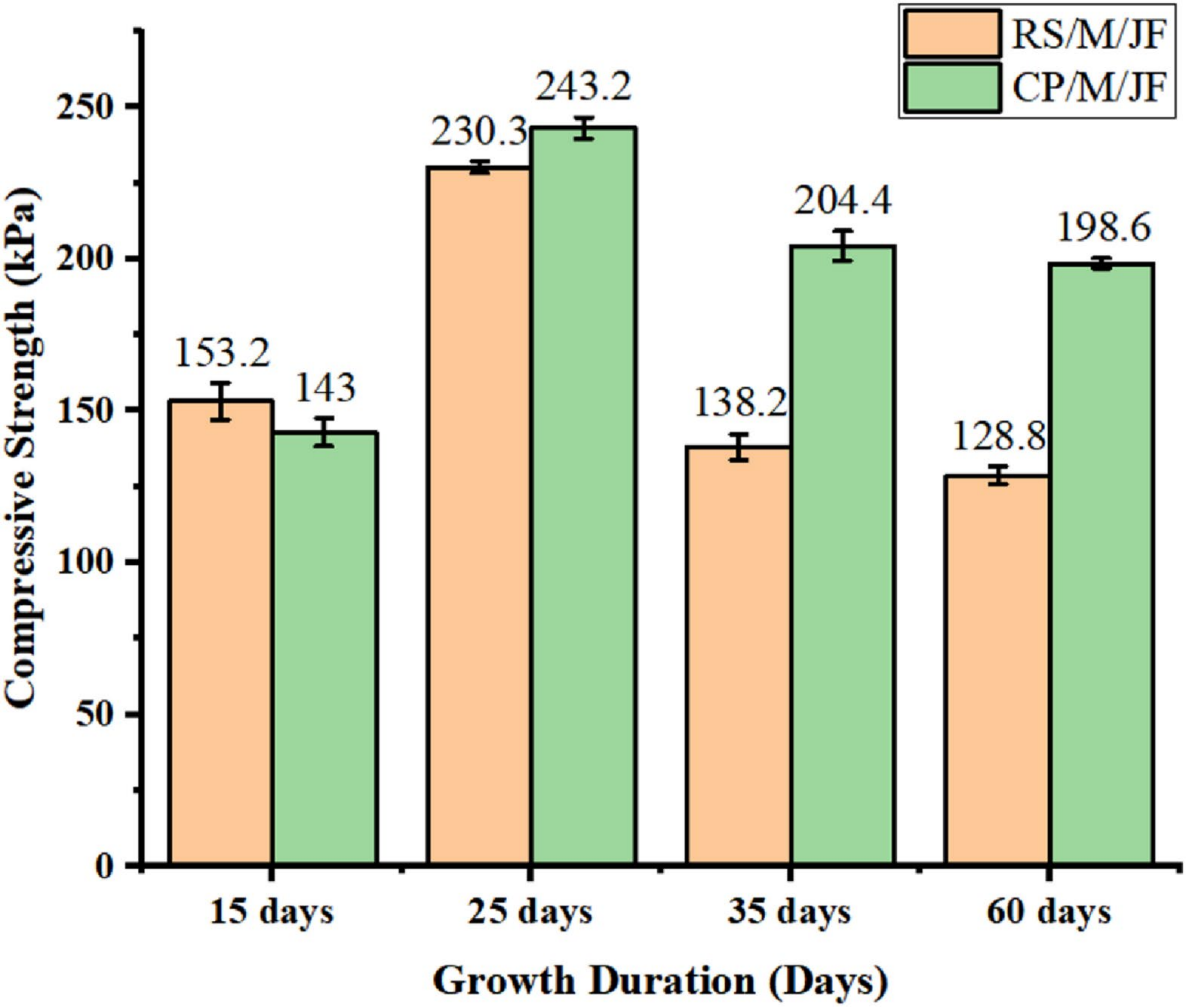
Variation in compressive strength of JF/RS/M and JF/CP/M composites with varied mycelium growth duration.

Additional tests were conducted after 60 days to measure the stability of these composites in real-life conditions. After controlled growth of 35 days, the composites were removed from the mycelium-friendly atmosphere, which impeded the further growth of the mycelium. This approach was followed to test the composite materials performance under normal conditions since these composites are being researched as a replacement for thermocol used in packaging. The evaluation showed a marginal loss of compressive strength compared to the reading obtained at 35th day but was very less as shown in Fig. 6. Compressive strength after 35 days was measured at 138 kPa and 204 kPa for RS/M/JF and CP/M/JF composites, respectively. After 60 days, these values slightly reduced to 129 kPa and 198 kPa, respectively, showing the stability of the composite materials even when exposed to normal conditions. These findings imply that mycelium-based composites can preserve adequate mechanical properties over time, making them a viable option for sustainable packaging.

### Flexural strength

**Fig. 7:**
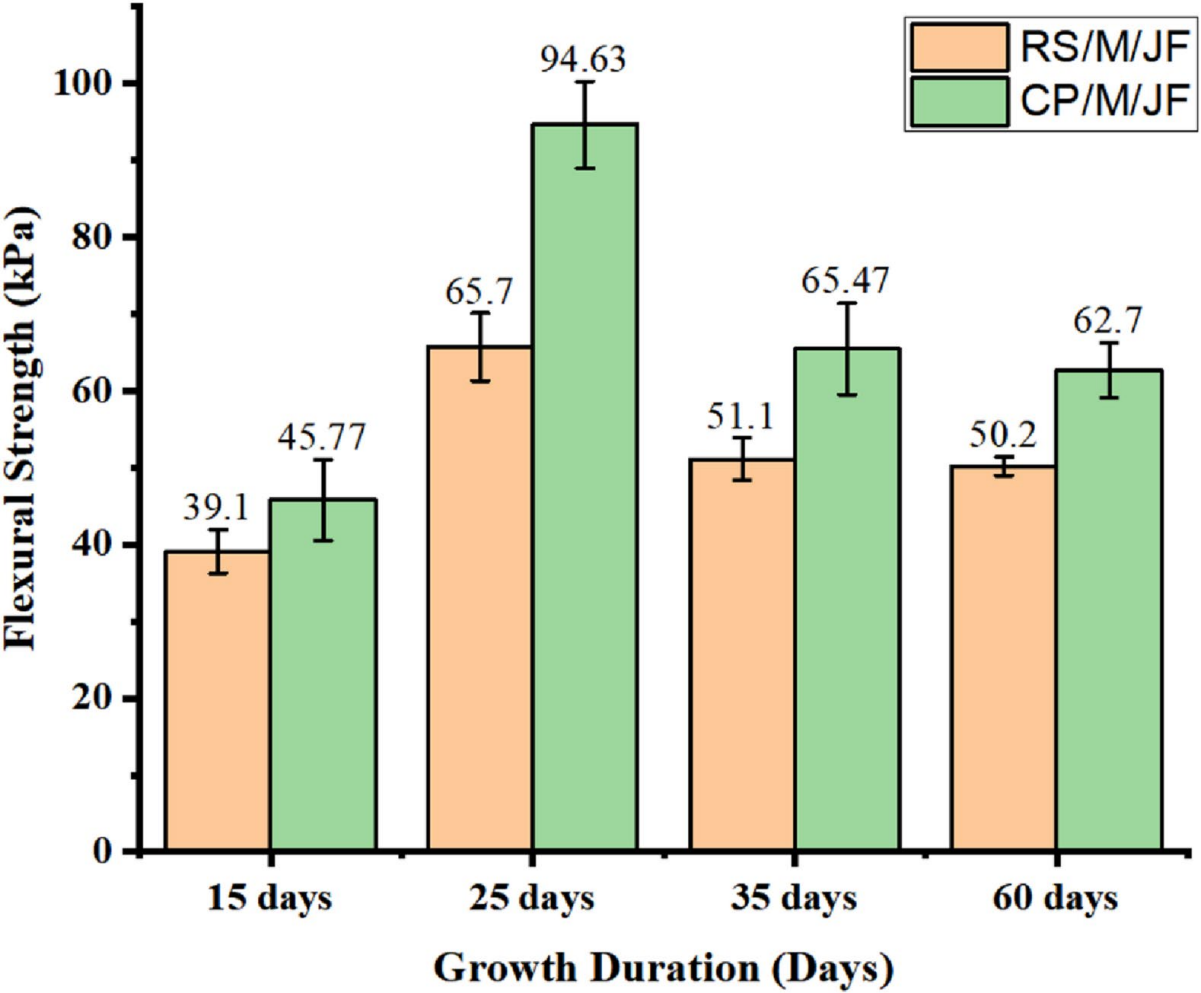
Variation in flexural strength of JF/RS/M and JF/CP/M composites with Mycelium growth at 15, 25, 35, and 60 days.

The results from the flexural tests on RS/M/JF and CP/M/JF composites during varied duration of mycelium growth are compiled in the Table 3. As we can see from the test results that the flexural strength of the RS/M/JF and CP/M/JF composites held for 15 days of growth duration is determined to be 39 kPa and 45.77 kPa, respectively. However, with increasing the growth duration to 25 days the flexural strength of the composites improved for 65.7 kPa and 94.63 kPa, which is 68% and 106% higher in comparison with initial 15 days grown composites as illustrated in the Fig. 7. It is also been seen that after 25 days of growth, the flexural strength of CP/M/JF is 45% higher than RS/M/JF. This increment is mainly due to the CP, which is in the form of powder. However, the RS reinforcements are in the form of whisker. Hence, during the mycelium growth and after the process of compaction and it is been expected that the densification of the composite due to the root growth was higher for the CP/M/JF.

**Table 3:** Results from flexural testing of the fabricated composites.

Mycelium-based composite	Growth duration	Flexural strength (kPa)				
		Sample 1	Sample 2	Sample 3	Average	Standard Deviation
JF/RS/M	15 days	35.4	39.5	42.3	**39.1**	2.83
	25 days	60.4	71.2	65.6	**65.7**	4.41
	35 days	51.6	54.2	47.5	**51.1**	4.76
	60 days	48.6	51.6	50.3	**50.2**	1.23
JF/CP/M	15 days	45.6	52.3	39.4	**45.8**	5.27
	25 days	95.3	101.2	87.4	**94.6**	5.65
	35 days	73.2	64.5	58.7	**65.5**	5.96
	60 days	65.2	65.3	57.6	**62.7**	3.6

RS: Rice straw M: JF: Jute fiber, CP: Coco-pit.

The significant increase in flexural strength observed after 25 days can likely be attributed to the complete development and maturation of the root system, which enhances the interfacial bonding and densification of the mycelium matrix. The differences in physical and chemical properties between sawdust and straw influence how the mycelium grows and the structural characteristics of the final composite. Sawdust, being composed of finer particles, allows for a denser packing of the substrate, which increases the density of the mycelium-based composite. On the other hand, straw is more fibrous and hollow, leading to a lower density when used by it. In addition, after 25 days of growing, the flexural strength of CP/M/JF is has been found to be 45% higher than that of RS/M/JF. This is mainly because of the powdered form of CP, while RS is in the form of whiskers. The particulate form of CP makes space-filling more efficient because of its lower aspect ratio (nearly spherical in shape), which causes a higher densification of the composite material. This further leads to better mechanical properties due to the enhanced density, which increases the strength of the matrix material of mycelium.

The improved mechanical performance of the cocopeat-based composites may be attributed to the finer particulate nature of cocopeat, which can promote a more uniform distribution of the mycelial network and enhanced interfacial bonding. However, this interpretation is qualitative, and further quantitative analysis, such as density and detailed microstructural characterization, is required to confirm this behavior.

It was seen that after 35 days of growth, the flexural strength began to drop for both RS/M/JF and CP/M/JF. This weakening may be associated with the fact that mycelia overgrowth leads to an end where excess entanglement occurs, thereby weakening the matrix structure. With increased growth of mycelia, it consumes all the nutrients, hence weakening the bond between the reinforcement and mycelium matrix. Moreover, with increased growth, partial decomposition of reinforcing fibers can occur, which will reduce their effective role in stress distribution. The balance between fiber reinforcement and mycelium growth is critical, given that excessive mycelial proliferation without sufficient structural reinforcement may result in compromising the mechanical integrity.

The results indicate that only minor variations were observed in compressive and flexural strength between 35-day and 60-day samples. Although slight variations were observed between the 35-day and 60-day mechanical properties, no definitive conclusion regarding statistical significance can be drawn due to the absence of replicate testing and statistical analysis. Future studies will include replicate testing and statistical analysis (e.g., standard deviation and significance testing) to validate the observed differences.

The reported mechanical properties represent the average values obtained from a limited number of specimens tested under each condition. A detailed statistical analysis, including standard deviation and significance testing, was not performed and will be considered in future studies to further validate the results.

Conventional petroleum-based packaging materials such as expanded polystyrene (EPS) exhibit higher mechanical strength and lower moisture sensitivity compared to mycelium-based composites. However, EPS is non-biodegradable and poses significant environmental concerns. In contrast, the developed mycelium-based composites, although lower in strength, offer advantages such as biodegradability, renewability, and reduced environmental impact. Therefore, these materials show potential for application in low-load and sustainable packaging systems where environmental considerations are critical.

## Conclusions

The developed mycelium-based composites demonstrate potential as sustainable alternatives for low-load packaging applications; however, further studies are required to comprehensively evaluate their performance before considering them as replacements for conventional petroleum-based materials. The strength reached its maximum value at 25 days of mycelium growth, while degradation happened due to overgrowing mycelium and depletion of nutrients. In comparison to other composites, it was observed that compressive and bending strength for the composite containing cocopeat was higher than for JF/RS/M due to its fine particulate size, which helped to increase matrix density. Compression and bending strengths of both composites remained almost unchanged when mycelium growth was stopped at 35 days and tests were carried out at 60 days, showing their long-term strength retention properties. The developed composites are composed of natural lignocellulosic materials, which are generally considered biodegradable; however, dedicated biodegradation and environmental impact assessments are required to quantitatively validate their sustainability. Biodegradable properties of mycelium composites along with good strength have established their efficacy to replace petroleum packaging materials.

The aging evaluation conducted up to 60 days provides a preliminary understanding of the short-term stability of the composites; however, extended studies over longer durations are required to fully assess their long-term performance for packaging applications. While the present study focuses on the mechanical and aging behavior of the developed composites, other important properties relevant to packaging applications, such as density, water absorption, thermal insulation, and impact resistance, were not investigated and will be addressed in future studies.

## Data Availability

The data supporting the findings of this study are not publicly available due to restrictions related to ongoing research but are available from the corresponding author upon reasonable request.
